# Prognostic value of secretory autophagosomes in patients with acute respiratory distress syndrome

**DOI:** 10.1186/s40364-023-00519-z

**Published:** 2023-09-07

**Authors:** Xue-cheng Dong, Xin-yi Xu, Yue-ru Huang, Xing-xing Zhu, Yi Yang, Wei Huang, Ling Liu

**Affiliations:** 1 Jiangsu Provincial Key Laboratory of Critical Care Medicine, Department of Critical Care Medicine, Zhongda Hospital, School of Medicine, Southeast University, Nanjing, 210009 Jiangsu China; 2 Department of Critical Care Medicine, Zhejiang Hospital, Hangzhou, 310013 Zhejiang China

**Keywords:** ARDS, Extracellular vesicles, Secretory autophagosomes, Liquid biopsy, Biomarker

## Abstract

**Background:**

Growing evidence supports that extracellular vesicles (EVs) in blood plasma and other body fluids may function as biomarkers for disease. We previously found that secretory autophagosomes (SAPs), a kind of EV, could exacerbate lung injury in mice. However, the clinical value of SAPs in acute respiratory distress syndrome (ARDS), the most severe form of lung injury, remains unknown. Our study investigated the prognostic value of secretory autophagosomes in ARDS.

**Methods:**

ARDS patients (n = 46) and controls (n = 8) were included in a prospective monocentric study. Bronchoalveolar lavage fluid (BALF) samples were collected from ARDS patients on the first day (Day 1) or the third day (Day 3) of enrollment and were collected from controls on Day 1. Gradient centrifugation was performed to isolate EVs. The size and concentration of EVs were characterized by nanoparticle tracking analysis (NTA). SAPs in EVs were characterized by flow cytometry, transmission electron microscopy, and western blot analysis, and the proportion of SAPs in EVs (PSV) was measured by flow cytometry. The association of SAPs with 28-day mortality was assessed.

**Results:**

On Days 1 and 3, the proportion of SAPs (SAPs%) in BALF was higher in patients with ARDS than in controls. On Day 3, the SAPs% was significantly higher in nonsurvivors than in survivors. In particular, a high SAPs% was associated with poor overall survival in ARDS patients. Furthermore, the combination of SAPs% and SOFA obtained a higher predictive value of ARDS outcome than PSV or SOFA alone.

**Conclusion:**

SAPs% in BALF is elevated in patients with ARDS and is associated with the risk of death in ARDS, suggesting that SAPs% may be a novel prognostic biomarker in ARDS.

**Supplementary Information:**

The online version contains supplementary material available at 10.1186/s40364-023-00519-z.

**To the editor:**

Acute respiratory distress syndrome (ARDS) is a devastating lung disorder characterized by the acute onset of severe hypoxemic respiratory failure with a high mortality of 30–40% [1, 2]. Effective stratification of ARDS patients would reduce mortality by optimizing therapy. The current widely used scoring systems, such as the lung injury severity score or SOFA (Sequential Organ Failure Assessment) score, successfully provide information on patient outcomes but fail to give consistent and accurate predictive estimates of the risk of death [3, 4]. Furthermore, these general severity scores do not have any pathophysiologic input [3]. Hallmarks of ARDS include uncontrolled alveolar inflammation (hyperinflammatory status), which contributes to lung injury [5, 6]. Therefore, stratification of ARDS patients with reliable biomarkers predictive of inflammation status and mortality would optimize treatment and guide personalized therapies.

Extracellular vesicles are a collective term for lipid bilayer-enclosed, cell-derived particles [7]. Notably, all types of immune cells participating in inflammation can secrete EVs, which in turn play multiple roles in inflammatory processes [8, 9]. We previously reported that secretory autophagosomes (SAPs) from LPS-stimulated macrophages could exacerbate lung injury in mice by transferring proinflammatory cytokines (IL-1β), indicating that SAPs have pivotal functions in ARDS pathophysiology [10]. In this study, we aimed to identify the clinical value of SAPs as a biomarker of the risk of subsequent mortality in ARDS patients by measuring the levels of SAPs in bronchoalveolar lavage fluid (BALF).

To evaluate the prognostic potential of SAPs, we first examined SAPs in the BALF of ARDS patients. Between November 2020 and January 2022, 122 patients were screened for eligibility, and 46 (37.7%) were included in the analysis. Among them, 46 and 24 patients had BALF drawn on Day 1 and Day 3, respectively (Fig. 1A). The reasons for exclusion are provided in Fig. 1A. The demographic and clinical characteristics of the ARDS subjects and non-ARDS controls are presented in Table 1. SAPs were isolated by differential centrifugation (Fig. 1B). Transmission electron microscopy (TEM) and nanoparticle tracking analysis (NTA) showed that SAPs were physically homogenous with a size peaking at 214.2 nm in diameter (Fig. 1C and D). Western blot and flow cytometry analyses of the characteristic membrane protein LC3II further confirmed the identity of SAPs (Fig. 1E and F). Thus, we successfully isolated EVs containing SAPs from BALF.

**Table 1:** Baseline characteristics of patients with ARDS and control subjects

Variables	ARDS (n = 46)	Controls (n = 8)	*P* value
Age (year)	58.50 [49.25-71.00]	37.50 [31.50-48.75]	**0.005**
Male, n (%)	32 (69.6)	8 (100.0)	0.095
Cause of ARDS, n (%)			
• Direct ARDS • Indirect ARDS	39 (84.8%) 7 (15.2%)		
Risk factor for ARDS, n (%)			
• Pneumonia • Sepsis • Pulmonary contusion • pancreatitis • Other	27 (58.6%) 3 (6.5%) 9 (19.5%) 3 (6.5%) 4 (8.9)		
Comorbidities, n (%)			
• Diabetes	5(10.9)	0(0.0)	1.000
• Hypertension	18(39.1)	4(50.0)	0.702
• Cardiovascular disease	7(15.2)	0(0.0)	0.540
• Cerebrovascular disease	25(54.3)	1(12.5)	0.052
• Urinary system disease	8(17.4)	0(0.0)	0.460
• Digestive system disease	13(28.3)	0(0.0)	0.201
APACHE II score	22.39 ± 7.15	6.50 ± 3.12	**< 0.001**
SOFA score	9.52 ± 3.66	1.5 ± 1.20	**< 0.001**
Mechanical ventilation setting			
• Tidal volume (ml)	400.00[342.50–420.00]	450.00[427.50–460.00]	**0.001**
• PEEP (cm H_2_O)	10.00[6.75-12.00]	5.00	**< 0.001**
• PaO_2_/FiO_2_ (mm Hg)	155.63 ± 63.03	321.43 ± 79.27	**< 0.001**
Laboratory variables			
• Plasma creatinine (umoL/L)	78.00[52.50–104.00]	79.50[70.25-84.00]	0.79
• White blood cell count (10^9^/L)	12.81[9.39–16.18]	6.56[5.39–7.56]	**0.003**
• Hemoglobin (g/L)	91.86 ± 25.75	148.63 ± 9.38	**< 0.001**
ICU stay (day)	18.00[8.00–33.00]	1	**< 0.001**

The data are presented as the mean ± standard deviation or as the median [interquartile range]. *P* values less than 0.05 were considered statistically significant. APACHE, acute physiology and chronic health evaluation; ARDS, acute respiratory distress syndrome; ICU, intensive care unit; PaO_2_/FiO_2_, the ratio of partial pressure of oxygen of fractional inspired oxygen; PEEP, positive end-expiratory pressure; SOFA, sequential organ failure assessment

**Fig. 1:**
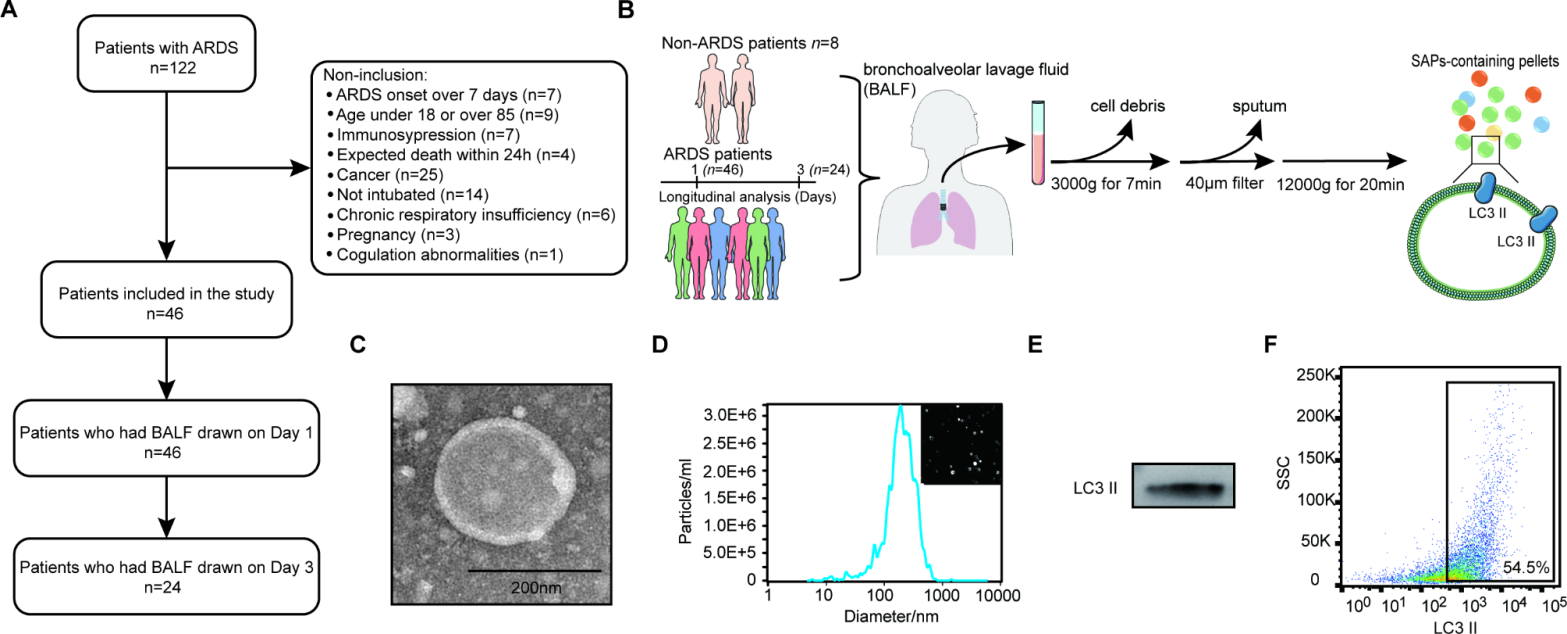
**Characterization of SAPs from BALF in ARDS patients**. (**A**) Flow chart of patients with ARDS included in the study. (**B**) The workflow of SAPs isolation. (**C**) The morphology of EVs was observed by TEM (scale bars = 200 nm). (**D**) The size distribution of EVs in ARDS patients was analyzed by NTA. The expression of SAPs marker (LC3) was analyzed by Western blotting (**E**) and flow cytometry (**F**)

We then compared SAPs in the ARDS and control groups. The absolute concentration of EVs from BALF was higher in ARDS patients on Day 1 than in the control group (Fig. 2A). Remarkably, the proportions of SAPs in EVs (SAPs%) of ARDS patients on Day 1 and Day 3 were both greater than those of controls, and there was no significant difference in the SAPs% between Day 1 and Day 3 (Fig. 2B). When dividing ARDS patients into two groups according to the initial site of infection, that is, intrapulmonary infection (ARDSp) or extrapulmonary infection (ARDSexp), we found that SAPs% was higher in ARDSp patients than in ARDSexp patients on Day 1 (Fig. 2C), whereas there was no significant difference on Day 3 (Fig. 2D), indicating that the SAPs% on Day 1 might reflect the site of infection. We next analyzed the cellular origin of SAPs by detecting cell type-specific markers and found that the proportion of macrophage-derived (CD68-positive) SAPs was dramatically elevated in ARDS patients (Fig. 2E), further supporting our previous work showing that macrophage-derived SAPs play key roles in ARDS pathogenesis. Overall, the higher level of SAPs in ARDS patients than in controls strongly suggests that SAPs might be associated with the prognosis of ARDS.

**Fig. 2:**
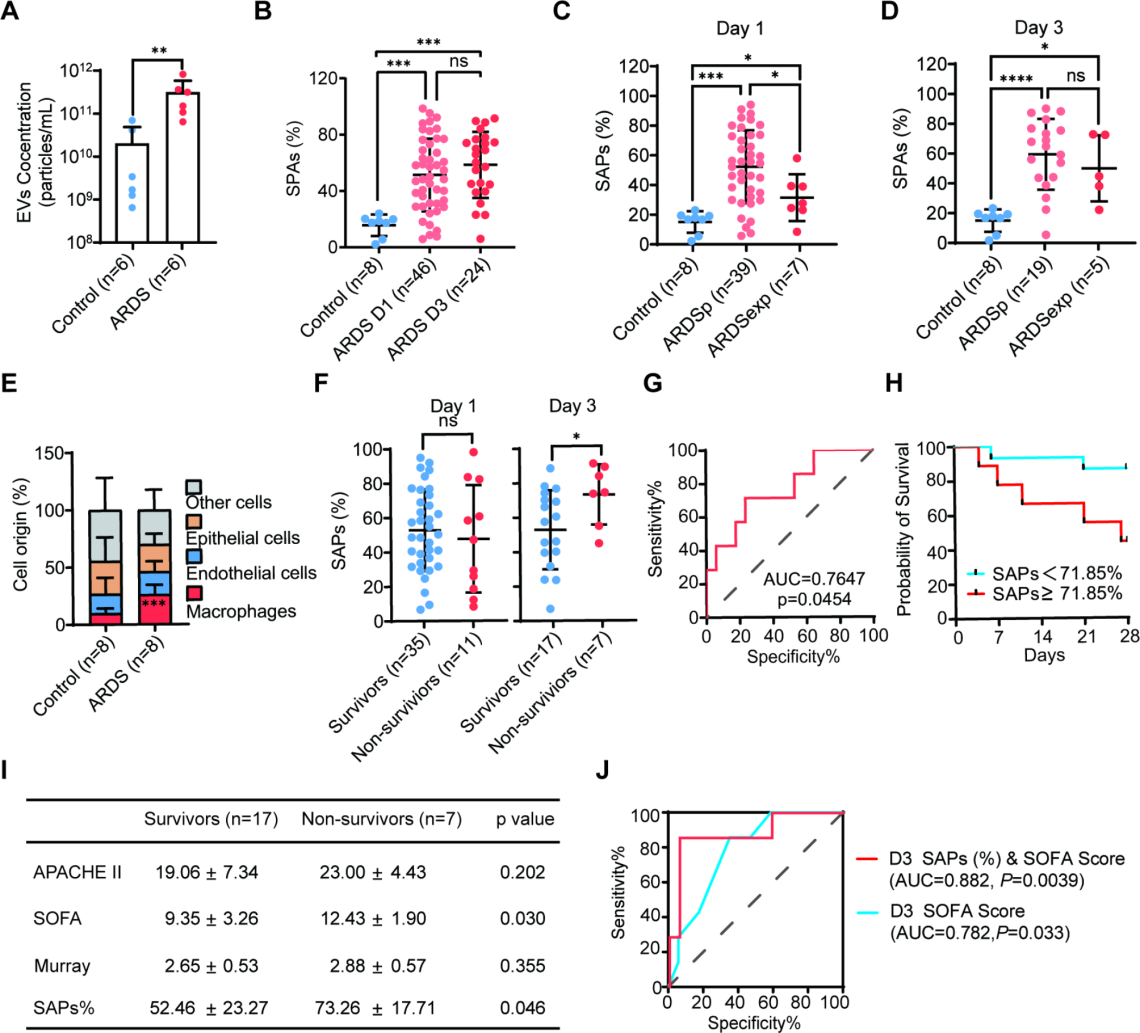
**SAPS% from BALF is associated with ARDS prognosis**. (**A**) The concentration of EVs in Day 1 ARDS patients (n = 6) and controls (n = 6). Data are normalized per 1mL BALF. Statistical significance is calculated using Mann-Whitney test; (**B**) SAPs% in ARDS patients on Day 1 (n = 46), Day 3 (n = 24) and controls (n = 8). Statistical analysis is calculated using one-way ANOVA; (**C**) BALF SAPs% in ARDSp patients (n = 39) and ARDSexp patients (n = 7) on Day 1 and in controls (n = 8). Statistical analysis is calculated using one-way ANOVA; (**D**) SAPS% in ARDSp patients (n = 19) and ARDSexp patients (n = 5) on Day 3 and in controls(n = 8). Statistical analysis is calculated using one-way ANOVA; (**E**) Cellular origin of SAPs in patients with ARDS (n = 8) and controls (n = 8). CD68 was used to identify macrophage derived EVs. CD31 and CD326 were used to identified EVs from endothelial and epithelial, respectively. (**F**) SAPs% in survivors (n = 35) and non-survivors (n = 11) on Day 1 and SAPs% in survivors (n = 17) and non-survivors (n = 7) on Day 3. Unpaired Student’s t test was used to calculate significance. (**G**) The ability of SAPs% on Day 3 to predict mortality in patients with ARDS. (**H**) The survival rate of patients stratified with a cutoff value of 71.85% for SAPs% on Day 3 was analyzed by Kaplan-Meier curves. (**I**) Comparison of clinical parameters according to the survival status on Day 3 using unpaired Student’s t test. (**J**) A combination of SAPs% and the SOFA score on Day 3 to predict the prognosis of ARDS. SAPs, secretory autophagosomes. SAPs%, the proportion of SAPs in EVs; Data are presented as mean ± SD in (**A**)-(**D**) and (**F**). *P < 0.05, **P < 0.005, ***P < 0.001, ****P < 0.0001

To explore the prognostic value of SAPs as biomarker carriers, we compared ARDS survivors to nonsurvivors. Surprisingly, although the level of SAPs was markedly increased in ARDS patients (Fig. 2B), there was no significant difference between ARDS survivors and nonsurvivors on Day 1 (Fig. 2F). Nevertheless, on Day 3, the SAPs% was significantly higher in nonsurvivors than in survivors (Fig. 2F). Thus, we mainly focused on the assessment of the prognostic value of SAPs% on Day 3. ROC analysis was performed to test the specificity and sensitivity of SAPs% on Day 3 to distinguish ARDS survivors and nonsurvivors. The AUC for SAPs% on Day 3 was 0.765 (95% CI 0.552–0.977, P = 0.045, Fig. 2G), and the optimal cutoff value was 71.85%. We used the optimal cutoff value to divide the patients into a high SAP group (SAPs ≥ 71.85%) and a low SAP group (SAPS%<71.85%). The Kaplan‒Meier survival curves showed that the high SAP group was at greater risk of death than the low SAP group (P = 0.028, Fig. 2H), indicating that the SAPs% on Day 3 could be a predictor of ARDS risk. We then sought to identify whether the SAPs% on Day 3 may be used in combination with other clinical variables to develop composite scores and obtain better efficiency. When comparing three well-established clinical scores, namely, APACHE II, SOFA and Murray, on Day 3, we found that only the SOFA score significantly differed between ARDS survivors and nonsurvivors (Fig. 2I). Thus, we combined the SAPs% on Day 3 with the SOFA score to perform ROC analysis and found that this combination achieved a higher AUC (0.882, 95% CI 0.715-1.000, P = 0.004) than the SOFA score alone (0.782, 95% CI 0.595–0.968, P = 0.033) (Fig. 2J).

In conclusion, we provide a novel EV-based biomarker to predict the risk of death of ARDS patients. The SAPs% from BALF was higher in patients with ARDS than in controls. Furthermore, macrophage-derived SAPs were dramatically elevated in ARDS patients. In particular, we showed that the BALF SAPs% on Day 3 had good predictive value for 28-day mortality in ARDS patients, especially when combined with the SOFA score. EV-based biomarker research is gaining momentum in clinical research and is rapidly gaining increased attention. Since EVs are highly heterogeneous, it would be interesting to identify different types of EVs as ARDS diagnostic and prognostic tools. However, the limitations of the study include the small sample size, lack of a validation cohort and failure to obtain samples at both time points for all patients, which limits its external validity and the ability to perform longitudinal analyses. Moreover, only three cell markers were evaluated, and more cell type-specific markers and the costaining of these markers with LC3 should be investigated in the future to explore the cell source of SAPs and their function in ARDS pathogenesis.

### Electronic supplementary material

Below is the link to the electronic supplementary material.

Supplementary Material 1

## Data Availability

The data that support the findings of this study are available from the corresponding author upon reasonable request.

## Abbreviations

EVs: Extracellular vesicles
ARDS: Acute respiratory distress syndrome
SAPs: Secretory autophagosomes
BALF: Bronchoalveolar lavage fluid
