# Supplementary material for: Prognostic value of secretory autophagosomes in patients with acute respiratory distress syndrome
Source: Biomark Res. 2023 Sep 7;11:79. doi: 10.1186/s40364-023-00519-z (PMC10483857; doi:10.1186/s40364-023-00519-z)
Supplement: Supplementary file 1 — Supplementary Material 1 [file 40364_2023_519_MOESM1_ESM.docx]

**Title：Prognostic value of secretory autophagosomes in patients with acute respiratory distress syndrome**

**Authors:** Xue-cheng Dong^1,2^, Xin-yi Xu^1^, Yue-ru Huang^1^, Xing-xing Zhu^1^, Yi Yang^1^, Wei Huang,^1^* Ling Liu^1^*

**Materials and Methods**

**Study population**

This prospective, single-center, observational cohort study was approved by the Clinical Ethics Committee of Zhong Da Hospital, Southeast University (approval number: 2021ZDSYLL215-P01). The patients who met the Berlin criteria for ARDS from the Department of Critical Care Medicine in Zhong Da Hospital were included from November 2020 to January 2022 (ClinicalTrials.gov number, NCT05101694 [ClinicalTrials.gov]). Informed consent was obtained from the legal representative of included patients. The exclusion criteria were as follows: Age younger than 18 years older or more than 85 years old, chronic respiratory insufficiency, ARDS onset over 7 days, immunosuppression, expected death within 24h, cancer, not intubated, pregnancy and coagulation abnormalities. The obstructive sleep apnea patients, who underwent uvulopalatopharyngoplasty surgery and were transferred to the Department of Critical Care Medicine in Zhong Da Hospital, were enrolled as controls. These patients were ventilated in ICU but without ARDS or infection. They stayed in ICU just 1 day. Demographic and baseline characteristics were collected from all study participants.

**SAPs extraction**

BALF was collected from ARDS patients on the first day (Day 1) or the third day (Day 3) of enrollment. BALF was collected from controls on Day 1.

BALF was collected as previously published.16 Briefly, after the fiberoptic bronchoscope was wedged into the selected bronchopulmonary segment, 20mL of saline was instilled on four separate occasions and the total volume (pooled aliquots) retrieved was greater than 30% of the total instilled volume. BALF was placed on ice and transported to the laboratory within 30 minutes. We extracted EVs containing SAPs according to the previously reported methods for isolating SAPs from macrophages.7 Briefly, BALF was centrifuged at 3000g for 7min at 4℃ to remove whole cells and debris, and the sputum in the BALF supernatants was removed through a 40 um filter (BS-40-XBS, Biosharp, China). The supernatants of BALF were further centrifuged at 12000g for 20min to harvest the SAPs-containing pellets. Finally, the pellets were resuspended in PBS and stored in a -80℃ freezer for further study. These processes are summarized in Figure 2a.

**Nanoparticle Tracking Analysis (NTA)**

NTA was used to visualize and quantitate EVs in suspension. Briefly, as described previously,7 17 isolated EVs were diluted with PBS to measure the particle size and concentration by ZetaView PMC 110 (Particle Metrix, Germany). NTA measurements were recorded and analyzed at 11 positions. The data was analyzed by ZetaView (version 8.05.12) and the system was calibrated using 100nm polystyrene particles.

**Transmission Electron Microscopy (TEM)**

The morphology of EVs was examined by TEM (HT7800, HITACHI, Japan). In brief, an isolated EV sample (5uL) was deposited on a formvar-carbon grid and incubated for 3-5min. The excess liquid was carefully dried with clean paper. Then, the absorbed EVs were negatively stained with 2% phosphotungstic acid (G1102, Servicebio, China) for 1-2 min and the excess fluid was drained. After the grid was dry at room temperature, the sample was observed by TEM.

**Western Blot Analysis**

The protein samples extracted from EVs with RIPA lysis buffer (KGP250, KeyGEN BioTECH, China) were resolved via 15% sodium dodecyl sulfate-polyacrylamide gel electrophoresis (P0461S, Beyotime, China) and transferred onto a polyvinylidene difluoride membrane with 0.2 um pore size (Immobilon-PSQ, Merck Millipore, Germany). The membrane was blocked with 5% skim milk in TBST for 1h and incubated overnight at 4℃ with the primary antibody anti-LC3B (3868, Cell Signaling Technology, USA). After washing with TBST buffer, the membrane was incubated with a horseradish peroxidase-conjugated anti-rabbit secondary antibody (7074, Cell Signaling Technology, USA) for 1h. The result was visualized by electrochemiluminescence detection kits (P0018FS, Beyotime, China).

**Flow Cytometry**

The proportion of SAPs in EVs (PSV) and the cellular origin of SAPs were measured by flow cytometry. In BALF, the Fc receptors of EVs after centrifugation at 12000g were blocked with the Fc receptor-blocking agent (130-059-901, Miltenyi Biotech, Germany) for 10min at 4℃. Next, EVs were incubated with the anti-LC3B (ab225383, Abcam, UK), anti-CD68 (AB_10805746, eBioscience, USA), anti-CD31 (303106, BioLegend, USA) and anti-CD326 (324206, BioLegend, USA) in the dark for 30min at 4℃. After washing with PBS, the samples were centrifuged at 12000g for 20min at 4℃. All data were acquired using a FACSCelesta multicolor flow cytometer (BD Biosciences, America) and analyzed by FlowJo (version 10.6.2, Tree Star, America).

**Statistical analysis**

All data were presented as mean ± standard deviation or median [interquartile range] as appropriate, and compared using the t test, the Mann-Whitney test or one-way analysis of variance. For comparison of PSV at different time points, the paired-samples t test or the Wilcoxon matched-pairs signed rank test was used, as appropriate. Categorical variables are reported as numbers and percentages and compared using the Fisher test. Receiver-operating characteristic (ROC) curve analysis was used to identify predictive values for surviving patients. The difference between AUC values was tested with the Delong test. Kaplan-Meier curves based on PSV levels and the log-rank test were used to compare survival rates.

All statistical analyses were two-tailed, and a P value less than 0.05 was considered significant. Analyses were performed using SPSS (version 25, IBM, USA), GraphPad Prism (version 8.4.3, GraphPad Software, USA), and MedCalc (version 20.100, MedCalc Software, Belgium).
